# Structural Entropy of Liver Histological Sections as an Integrative Index of Tissue Disorganization in Constant-Light-Induced Hepatic Remodeling with Aging-Associated Features

**DOI:** 10.3390/biomedicines14091968

**Published:** 2026-08-31

**Authors:** David A. Areshidze, Nikita S. Gladyshev, Maria A. Kozlova, Anna I. Anurkina

**Affiliations:** Avtsyn Research Institute of Human Morphology, B.V. Petrovsky Russian Scientific Center of Surgery, 141076 Moscow, Russia

**Keywords:** constant light exposure, dark deprivation, liver aging, structural entropy, entropy, circadian rhythms, melatonin, cellular senescence, steatosis, mitochondrial dysfunction, digital pathology

## Abstract

**Objective:** To evaluate whether structural entropy of liver histological sections reflects tissue disorganization in a rat model of constant light exposure (dark deprivation) and to explore its associations with morphofunctional parameters of hepatic remodeling. **Methods:** Male Wistar rats (*n* = 120) were divided into a control group (12:12 h light/dark) and a constant light group (24 h/day, 3 months). Biochemical, histological, immunohistochemical (p16, p21, p53, BMAL1, CLOCK, PER2, Ki-67) and ultrastructural parameters were assessed. Shannon entropy was calculated on hematoxylin and eosin-stained sections using a graph-based approach based on nuclear spatial distribution and size heterogeneity. **Results:** Constant light exposure caused a >2-fold decrease in serum melatonin, suppression of BMAL1 and CLOCK expression (3.7- and 3.5-fold, respectively), a rise in PER2 (+29.8%), and marked increases in p16 (23.8-fold), p21 (6.7-fold), p53 (+80.9%), steatosis, hyperglycemia, and mitochondrial dysfunction (decreased cristae count, reduced circularity index). Entropy increased by 21.4% and showed strong correlations with steatosis score (r = 0.93), p53-positive cells (r = 0.88), PER2-positive cells (r = 0.86), and an inverse correlation with melatonin (r = −0.81). Partial correlations adjusted for treatment revealed that entropy remained associated with local morphological and ultrastructural parameters but not with systemic biochemical markers, suggesting that entropy primarily reflects tissue-level remodeling. An exploratory regression model, albeit with substantial multicollinearity, explained 87.5% of entropy variance, with steatosis and circadian markers as major contributors. **Conclusion:** Constant light exposure induces hepatic alterations that share multiple features with aging-associated phenotypes, including steatosis, cellular senescence, mitochondrial dysfunction, and circadian disruption. Structural entropy is an exploratory quantitative index of tissue disorganization that correlates with key morphofunctional parameters of hepatic remodeling. However, because the study lacked aged comparator groups, melatonin replacement, recovery models, and independent validation, entropy should not be considered a validated biomarker of biological liver age. Its potential applicability in digital pathology and geroprotective research requires confirmation in future studies with appropriate experimental designs.

## 1. Introduction

Aging is a universal, multifactorial process characterized by progressive accumulation of damage, decline in physiological functions, and increased vulnerability to disease [1]. The free radical theory, proposed by Harman in 1956, highlighted reactive oxygen species (ROS) as key drivers of cellular damage [1]. Although this theory does not fully account for all aging mechanisms, oxidative stress remains a central factor in age-associated pathologies, particularly in metabolically active organs such as the liver [2].

A critical link between aging, metabolism, and circadian regulation is melatonin, a pineal hormone secreted predominantly during darkness. Melatonin secretion declines with age, paralleling the degradation of circadian rhythmicity [3]. Aging is also accompanied by chronic low-grade inflammation (“inflammaging”) [4], and melatonin has been proposed as a candidate molecule capable of mitigating some age-related changes through its antioxidant and chronobiotic properties [4,5]. In the liver, melatonin has been shown to attenuate steatosis, inflammation, and mitochondrial dysfunction in various experimental models [5,6,7]. However, most of these studies employed pharmacological supplementation or genetic models, and the causal contribution of endogenous melatonin decline to hepatic aging phenotypes remains incompletely defined.

An important external factor that can precipitate melatonin deficiency and circadian disruption is alteration of the photoperiod. In modern environments, constant artificial lighting (“light pollution”) is increasingly recognized as a risk factor for metabolic and age-associated disorders [8,9,10,11]. Prolonged exposure to light at night suppresses pineal melatonin production and desynchronizes central and peripheral circadian oscillators [12]. Importantly, photic cues are among the most powerful entraining signals for circadian rhythms; they act not only via the melatonin axis but also through direct retinohypothalamic projections to the suprachiasmatic nucleus, which subsequently modulates autonomic nervous system outflow to peripheral organs, including the liver [13]. Thus, constant light exposure likely disrupts multiple interrelated pathways, and melatonin suppression should be viewed as one component of a broader chronodisruption syndrome rather than the sole mediator of hepatic effects.

The liver is particularly susceptible to circadian dysfunction because it possesses its own autonomous circadian clocks and expresses clock genes that regulate metabolism, detoxification, and regenerative capacity [13]. Desynchronization of these rhythms or dysregulation of clock gene expression contributes to steatosis, inflammation, fibrosis, and even carcinogenesis [13]. Previous studies have shown that constant light exposure in rats leads to reorganization of circadian rhythms of hepatic parameters, hepatocyte hypertrophy, lipid accumulation, and mitochondrial dysfunction [14,15]. These changes overlap with those observed in aging, including cellular senescence, impaired organelle turnover, and metabolic dysregulation [15]. However, whether this constellation of changes represents true accelerated aging or rather a stress-induced remodeling that phenocopies certain aging features remains an open question, as direct comparisons with naturally aged animals have not been performed.

In this context, the concept of structural entropy—derived from information theory—has gained attention as a potential integrative measure of tissue disorganization. Shannon entropy quantifies the uncertainty or heterogeneity of a system; applied to histological images, it can capture spatial and morphological complexity [16,17,18,19,20]. Studies have shown that entropy of network interactions increases with age in several organs, including the liver, reflecting loss of cellular identity and increased randomness [16]. Kayser and colleagues established the theoretical and methodological basis for using entropy in quantitative pathology, demonstrating its utility in characterizing tissue heterogeneity in neoplasms [20,21,22,23]. More recently, structural entropy has been proposed as a marker of age-related disorganization, although its relationship with specific molecular, ultrastructural, and metabolic hallmarks of aging under conditions of chronodisruption has not been systematically explored.

Given the background above, the present study was designed with an **exploratory** aim: to evaluate whether structural entropy of liver histological sections correlates with a panel of morphofunctional parameters in rats exposed to constant light, and to assess its potential as an integrative index of tissue disorganization that reflects aging-associated hepatic remodeling. We examined biochemical, histological, immunohistochemical (senescence markers p16, p21, p53; circadian proteins BMAL1, CLOCK, PER2; proliferation marker Ki-67), and ultrastructural parameters, and calculated graph-based Shannon entropy from H&E-stained sections. We also performed partial correlation analyses to disentangle associations driven by the experimental treatment from those reflecting local tissue changes.

Importantly, we do not claim to have validated entropy as a biomarker of biological liver age. The study lacks naturally aged comparator groups, melatonin-replacement or recovery groups, and independent validation cohorts. Therefore, our findings are hypothesis-generating rather than confirmatory. We present this work as a foundation for future investigations that will require direct comparisons with physiological aging, interventional designs, and prospective validation. With these caveats, we believe that the present dataset provides useful insights into the relationship between chronodisruption, hepatic remodeling, and tissue entropy, and may inform the development of quantitative tools for digital pathology.

## 2. Materials and Methods

### 2.1. Object of Study

This study was conducted on 120 male rats of Wistar outbred stock. At the beginning of the experiment, animals were 3 months old. Animals were obtained from the Research and Production Enterprise “Laboratory Animal Nursery ‘Pushchino’” (Branch of the FSBSI Shemyakin and Ovchinnikov Institute of Bioorganic Chemistry of the Russian Academy of Sciences). All animals were kept under standard vivarium conditions, with ad libitum access to drinking water and briquetted food. Initially, all rats were kept under natural daylight. The care of the animals and the experimental procedures were conducted in accordance with the European Convention for the Protection of Vertebrate Animals used for Experimental and other Scientific Purposes (Strasbourg, 18 March 1986). Ethical approval for the research was granted by the Bioethical Committee of the Avtsyn Research Institute of Human Morphology of FSBSI “Petrovsky National Research Centre of Surgery” (Protocol No. 5, 23 May 2025).

### 2.2. Study Design

At the age of 3 months, the rats were divided into 2 equal groups. The sample size (*n* = 60 per group) was determined based on a power calculation to detect statistically significant differences in the primary outcome measures (entropy, senescence markers) with 80% power and a two-sided significance level of α = 0.05, adjusted for multiple comparisons. Effect size estimates and standard deviations were derived from our pilot experiments and from published studies using the dark deprivation model [14]. This sample size also provided sufficient observations for correlation and regression analyses involving 33 variables (recommended minimum ratio of at least 3–5 observations per predictor).

Animals were randomly assigned to the control and experimental groups using simple randomisation with a random number generator (Microsoft Excel, RAND function). Each animal received a unique identification number, and group allocation was determined randomly. The randomisation procedure was performed by an independent researcher who was not involved in subsequent experimental manipulations or outcome assessments. Allocation concealment was ensured by coding the group identities until all experiments and measurements were completed.

**Control group (C, *n* = 60):** Animals were kept under a fixed light regime (light/dark ratio of 12:12 h with lights on at 08:00 and off at 20:00) for 3 months.

**Constant light group (LL, *n* = 60):** Animals received constant lighting (24 h/day) for 3 months.

All animals were provided with standard laboratory chow and water ad libitum throughout the experiment.

Throughout the 3-month experimental period, animals were monitored daily for general behaviour, motor activity, coat condition, food and water intake, and responses to external stimuli. Body weight was recorded weekly using an electronic balance (accuracy 0.1 g). Pre-established humane endpoints for early removal from the experiment included: loss of more than 20% of initial body weight, obvious signs of distress (apathy, refusal to eat, impaired coordination), visible tumours, bleeding or deep wounds, and signs of infection. No animal met any of these humane endpoints during the study; all rats completed the experimental protocol.

**Study design limitations acknowledged:** The present study did not include naturally aged comparator groups (e.g., 18- and 24-month-old rats), melatonin-replacement groups, or photoperiod recovery groups. Therefore, the study is **exploratory and hypothesis-generating** rather than confirmatory. These limitations are addressed in the Discussion and Limitations sections.

### 2.3. Sample Collection and Euthanasia

At the end of the experimental period, animals were euthanized by decapitation under light ether anaesthesia. Euthanasia was performed as follows: anaesthesia was induced by placing rats in an airtight chamber saturated with diethyl ether (5% *v*/*v*) until the surgical stage (loss of reflexes, deep breathing). Euthanasia was performed by rapid decapitation using a small animal guillotine (Harvard Apparatus 55 0012, Holliston, MA, USA) while the animal remained inside the chamber under continuous ether supply to maintain unconsciousness. Death was confirmed by the absence of heartbeat and respiration.

**Rationale for decapitation:** Decapitation is preferred over chemical euthanasia in studies of this type for two reasons. First, chemical euthanasia methods modulate measurements of biochemical parameters in blood plasma and serum compared with normal values (e.g., CO_2_ causes acidosis, and potassium chloride prevents analysis of serum potassium levels); moreover, anaesthetic agents can directly affect tissue viability or parameters. Chemical agents may directly damage tissues (e.g., intraperitoneal alcohol and intraperitoneal pentobarbital both diminish the tinctorial qualities in histological sections). Decapitation is also suitable when specimens are taken for electron microscopic analysis. Of all euthanasia methods, decapitation is the most consistent with these requirements [24,25,26].

Blood was collected into tubes with heparin for plasma separation. Liver tissue was rapidly excised, weighed, and processed for histological, immunohistochemical, and electron microscopic examination. After sacrifice, evisceration was performed. To account for circadian fluctuations, animals were euthanized at 09:00, 15:00, 21:00, and 03:00 (*n* = 15 per time point per group).

### 2.4. Biochemical Analysis

Plasma melatonin concentration was determined using a commercially available enzyme immunoassay kit “ELISA Kit for Melatonin” (CEA908Ge, Cloud Clone Corp., Katy, TX, USA) according to the manufacturer’s instructions, using an ELISA 200 analyser (Yantai ADC, Beijing, China).

Biochemical parameters, including glucose, aspartate aminotransferase (AST), alanine aminotransferase (ALT), total protein, albumin, cholesterol, triglycerides, lactate dehydrogenase (LDH), and alkaline phosphatase (ALP), were measured using an automated biochemical analyser (Cormay Accent 200, Cormay, Warsaw, Poland) with standard reagent kits (Cormay, Warsaw, Poland).

### 2.5. Histological and Morphometric Analysis

Liver fragments were fixed in 10% neutral buffered formalin, dehydrated in ascending concentrations of ethanol, and embedded in paraffin. Sections of 5 μm thickness were prepared and stained with haematoxylin and eosin (H&E) for general morphology assessment, and with Masson’s trichrome for connective tissue evaluation.

Morphometric analysis was performed using a Leica DM2500 microscope (Leica Microsystems, Wetzlar, Germany) equipped with a Leica DFC7000 T digital camera and Leica Application Suite X (LAS X) v. 4.13 image analysis software. The following parameters were measured in at least 50 fields of view per animal:

- Cross-sectional area of hepatocyte nuclei (S nucleus, μm^2^)
- Cross-sectional area of hepatocytes (S hepatocyte, μm^2^)
- Nuclear-to-cytoplasmic ratio (N/C ratio)—calculated as nuclear area divided by cytoplasmic area (cytoplasmic area = hepatocyte area—nuclear area)
- Proportion of binuclear hepatocytes (%)—determined by counting at least 1000 hepatocytes per animal
- Ploidy of hepatocytes—total liver preparations were used to obtain imprints, which were then stained with fuchsin-sulphurous acid according to the Feulgen method. Hepatocyte ploidy was calculated in ploidy units relative to the optical density of the stained diploid nuclei of small lymphocytes [27,28].
- Proportion of hepatocytes with fatty degeneration (%)—assessed in H&E-stained sections
- Severity of steatosis—evaluated semi-quantitatively using a 4-point scale: 0—no steatosis, 1—mild (<30% of hepatocytes), 2—moderate (30–60%), 3—severe (>60%) [29,30]

**Glycogen and lipid content:** Glycogen content was assessed by the PAS reaction (periodic acid–Schiff) with diastase control. Lipid content was assessed by Oil Red O staining on frozen sections. Optical density of glycogen and lipid staining was measured using ImageJ 4.0. software (NIH, Bethesda, MD, USA) in at least 50 fields per animal.

**Data aggregation:** For each animal, all morphometric measurements were averaged across fields to obtain a single value per parameter. Thus, the animal (*n* = 120) was the unit of statistical analysis.

### 2.6. Immunohistochemical Analysis

Immunohistochemical staining was performed on paraffin sections using the streptavidin–biotin–peroxidase method. Sections were deparaffinised, rehydrated, and subjected to heat-induced antigen retrieval in citrate buffer (pH 6.0). Endogenous peroxidase activity was blocked with 3% hydrogen peroxide. Sections were incubated overnight at 4 °C with primary antibodies against the following markers. Immunohistochemical staining was performed using the following primary antibodies: anti-p16 (rabbit polyclonal, Abcam ab108349, 1:200), anti-p21 (rabbit polyclonal, Abcam ab109520, 1:150), anti-p53 (mouse monoclonal, Abcam ab1101, 1:100), anti-BMAL1 (rabbit polyclonal, Novus Biologicals NB100-2288, 1:250), anti-CLOCK (rabbit polyclonal, Abcam ab3517, 1:200), anti-PER2 (rabbit polyclonal, Abcam ab180655, 1:150), and anti-Ki-67 (rabbit monoclonal, Abcam ab16667, 1:200) [31,32].

After washing, sections were incubated with appropriate biotinylated secondary antibodies, followed by streptavidin-horseradish peroxidase complex. Visualization was performed using 3,3′-diaminobenzidine (DAB) as chromogen. Sections were counterstained with haematoxylin. For negative controls, primary antibodies were omitted. Sections in which primary antibodies were replaced with phosphate-buffered saline served as controls.

**Quantification:** The expression index was calculated as the percentage of cells with positive nuclear staining relative to the total number of counted cells. At least 1000 cells were counted per section per animal. For each animal, the percentage of positive cells was averaged across three sections. The investigator performing the quantification was blinded to group allocation.

### 2.7. Electron Microscopy and Morphometric Analysis of Organelles

Liver samples (approximately 1 mm^3^) were fixed with 2.5% glutaraldehyde in phosphate buffer (pH 7.4), post-fixed in 1% osmium tetroxide (OsO_4_), dehydrated in ethanol, contrasted with 1% uranyl acetate in 70% ethanol during dehydration, and embedded in an Epon–Araldite mixture according to standard procedures. Ultrathin sections (approximately 70 nm) were obtained on a UC Enuity ultramicrotome (Leica Microsystems CMS GmbH, Wetzlar, Germany), additionally contrasted with Reynold’s lead citrate, and examined using a field emission scanning electron microscope HIMERA EM50X (CIQUTEK, Hefei, China). Images were captured digitally.

**Sampling and analysis:** For analysis, uniform areas were selected from the intermediate zone of the liver lobules (perivenular and periportal areas were avoided to reduce zonal heterogeneity). The analysis was performed in 10 random fields of view at ×10,000 magnification (area 25 μm^2^ each) to determine the numerical density of mitochondria and the sizes of hepatocytes and their nuclei, and in 10 random fields of view at ×20,000 magnification (area 6.25 μm^2^ each) for micromorphometry of mitochondria and the Golgi complex [33,34].

The dissector method was used for stereometric studies [34]. All measurements were performed on cross-sections of mitochondria. The following parameters were assessed:

- **Numerical density (n/μm^2^):** number of mitochondria per unit cytoplasmic area
- **Cross-sectional area (μm^2^)**
- **Perimeter (μm)**
- **Number of cristae per mitochondrion**
- **Area-to-perimeter ratio**
- **Circularity index:** calculated as 4π × Area/Perimeter24*π* × Area/Perimeter2 (a value of 1.0 indicates a perfect circle; lower values indicate more elongated or irregular shapes)

For each animal, all ultrastructural parameters were averaged across the 20 fields to obtain a single value per parameter.

### 2.8. Definition and Calculation of Structural Entropy (Graph-Based Shannon Entropy)

The entropy measure used in this study is **not a conventional image texture entropy** (e.g., based on pixel intensity distributions) but rather a **graph-based entropy** of the spatial and size distribution of hepatocyte nuclei. The rationale is that age-related and stress-induced tissue disorganization manifests as increased heterogeneity in nuclear size and spatial arrangement. The calculation proceeded as follows:

Step 1: Image acquisition

Liver sections stained with H&E were digitized using a Leica DM2500 microscope with a Leica DFC7000 T camera at ×400 magnification (field of view approximately 250 × 250 µm, resolution 0.12 µm/pixel). Images were saved as uncompressed TIFF files (2048 × 2048 pixels, 8-bit).

Step 2: Preprocessing

Colour deconvolution (scikit-image library, Python 3.8) was applied to isolate the haematoxylin (nuclear) channel from the eosin (cytoplasmic) channel. Only the haematoxylin channel (8-bit grayscale) was used for further analysis. Background subtraction was performed using a rolling-ball algorithm (radius = 50 pixels). Noise reduction was applied using a median filter (3 × 3 kernel).

Step 3: Segmentation and object filtering

Images were binarized using Otsu’s automatic thresholding. Overlapping nuclei were separated using a watershed algorithm (scikit-image). Objects with area <20 µm^2^ or >120 µm^2^ were excluded (non-hepatocyte nuclei, such as endothelial or Kupffer cells, and artefacts). Central veins, portal tracts, large vessels, and necrotic or fatty areas were manually excluded from all fields using a region-of-interest tool.

Step 4: Sampling

From each animal, 10 fields were randomly selected using systematic random sampling with a grid. The same number of regions of interest was used per animal.

Step 5: Feature extraction

For each segmented nucleus, the following features were recorded:

- Cross-sectional area (A, µm^2^)
- Centroid coordinates (x, y)

Step 6: Construction of the Minimum Spanning Tree (MST)

Using the R package igraph (v. 1.5.0), the MST was constructed on the set of nuclear centroids. Edge weights were defined as the Euclidean distance between centroids. The MST connects all nuclei such that the total edge length is minimized and no cycles are formed. Each edge ee connects two nuclei i and j and is characterized by:

- Euclidean distance dij
- Area difference ΔAij = ∣Ai − Aj∣

Step 7: Probability definition

The distribution of ΔAij values across all edges was divided into K = 10 equal-frequency bins (i.e., bins with approximately equal numbers of edges). The probability pk for bin k was defined as:

pk = Nedgesnk

where:

- nk is the number of edges with ΔA values falling into bin k
- Nedges is the total number of edges in the MST (Nedges = Nnuclei − 1)

Step 8: Entropy calculation

Shannon entropy was then computed as:

H = −k = 1∑Kpklog2(pk)

Step 9: Rationale and interpretation

Higher entropy values indicate greater heterogeneity in nuclear size differences between adjacent cells. In a histologically uniform tissue, most adjacent nuclei have similar sizes, so most edges fall into a narrow range of ΔA, yielding low entropy. In a disorganized tissue (e.g., with cellular hypertrophy, senescence, or steatosis), nuclear sizes vary widely, and ΔA values are more uniformly distributed across bins, yielding higher entropy.

Step 10: Robustness checks

To ensure the stability of the entropy measure, the calculation was repeated with:

- K = 5, 10, 15, 20 bins (results were highly consistent; Cronbach’s α > 0.95 across bin numbers)
- Edge length dij instead of ΔAij as the weighting variable (results correlated with the primary measure at r > 0.92)

Step 11: Final entropy value

The final entropy value for each animal was the mean of the entropy values computed from the 10 fields. All image processing and entropy calculations were performed using custom Python scripts (available at [repository link]). The investigator performing the analysis was blinded to group allocation.

### 2.9. Statistical Analysis

Statistical analysis was performed in R (v. 4.4.2) using the packages lmtest, car, and MASS [35,36,37]. The analysis included only male rats from the control group and the constant light exposure group.

**Unit of analysis:** The animal was the sole unit of statistical analysis. For every parameter, measurements from multiple fields, sections, or cells were aggregated to a single value per animal before any statistical test:

- **Histological and morphometric parameters** (nuclear area, hepatocyte area, N/C ratio, binuclear cell proportion, ploidy) were averaged from at least 50 fields of view per animal.
- **Immunohistochemical markers** (p16, p21, p53, BMAL1, CLOCK, PER2, Ki-67) were averaged from at least 1000 cells counted per animal.
- **Mitochondrial ultrastructural parameters** were averaged from 10 electron microscopy fields per animal.
- **Entropy** was averaged from 10 histological fields per animal.
- **Biochemical parameters** were measured once per animal and required no aggregation.

Thus, *n* = 120 represents 120 independent animals, not 120 fields or cells. All reported *p*-values, correlations, and regression coefficients are based on animal-level data. This eliminates pseudoreplication as a concern.

**Data presentation:** Continuous variables are presented as mean ± standard deviation (M ± SD). Ordinal histological scores are presented as median [Q1; Q3].

**Group comparisons:** Between-group comparisons were performed between the control and constant light groups. Continuous variables were analysed using a univariable linear model with treatment group as the explanatory variable. Ordinal histological scores were compared using the Wilcoxon rank sum test. For each outcome, the between-group difference, 95% confidence interval, test statistic, *p*-value, and adjusted q-value were reported. Effect sizes were calculated as Hedges’ g for continuous variables and Cliff’s delta for ordinal variables.

**Multiple testing correction:** To account for multiple testing, *p*-values were adjusted using the Benjamini–Hochberg false discovery rate (FDR) procedure within predefined biological domains. Adjusted *p*-values are reported as q-values. Results were considered statistically significant at q < 0.05.

**Correlation analysis:** Associations between Shannon entropy and morphofunctional parameters were assessed using correlation analysis. Pearson correlation coefficients were calculated for continuous variables, whereas Spearman correlation coefficients were calculated for ordinal histological scores. Correlation coefficients, sample size, 95% confidence intervals (when available), *p*-values, and Benjamini–Hochberg adjusted q-values were reported.

**Partial correlation analysis:** To assess associations between Shannon entropy and morphofunctional parameters not explained solely by the experimental exposure, partial correlation analysis adjusted for treatment group was performed. For continuous variables, partial Pearson correlations were calculated. For ordinal variables, rank transformation was applied before residualization, and partial Spearman correlations were reported. Partial correlation coefficients, 95% confidence intervals, *p*-values, and q-values were reported.

**Exploratory multivariable regression model:** An exploratory multivariable linear regression model was constructed to describe the combined association of morphofunctional parameters with Shannon entropy. Shannon entropy was used as the dependent variable. Candidate predictors included variables significantly associated with entropy in univariable correlation analysis and anthropometric parameters. The final model was selected using bidirectional stepwise selection based on the Akaike Information Criterion (AIC). Regression coefficients, standard errors, standardized beta coefficients, 95% confidence intervals, t-statistics, *p*-values, q-values, partial R^2^ values, and variance inflation factors (VIFs) were reported.

**Model performance and diagnostics:** Model performance was evaluated using R^2^, adjusted R^2^, root mean square error (RMSE), mean absolute error (MAE), residual standard error, F-statistic, AIC, and Bayesian Information Criterion (BIC). Regression assumptions were assessed using residual diagnostics: normality of residuals (Shapiro–Wilk test), homoscedasticity (Breusch–Pagan test), residual independence (Durbin–Watson test), and linearity (graphical assessment using residual and observed-versus-predicted plots).

**Interpretational caveat for the regression model:** The multivariable regression model was developed using AIC-based stepwise selection **and is presented as an exploratory descriptive model, not as a validated predictive or causal tool.** The high R^2^ (0.875) was obtained on the same dataset used for model development; independent validation in a separate cohort is required. Because variance inflation factors were high (VIF > 100 for several predictors), individual coefficients should not be interpreted as independent or causal effects. The model serves only to illustrate the combined association of interrelated morphofunctional parameters with entropy, consistent with its integrative nature.

**Blinding:** To minimise observer bias, all histological, immunohistochemical, and ultrastructural slides were coded with random numerical identifiers, and the investigator performing microscopic evaluation and morphometry was blinded to group allocation throughout the analysis (blinding of outcome assessment). Entropy calculation was performed using an automated algorithm, eliminating any experimenter influence. Biochemical analyses were performed on an automated analyser, and the operator was not informed of the group distribution. Group decoding was performed only after all measurements were completed and the database was finalised.

**Statistical significance:** All statistical tests were two-sided. Results were considered statistically significant at q < 0.05 after FDR correction. For the exploratory regression model, uncorrected *p*-values are also reported for transparency, with explicit acknowledgment that this model is descriptive rather than confirmatory.

## 3. Results

### 3.1. Daily Mean Values of the Studied Parameters

This section presents the results of a comparative analysis of morphofunctional parameters of the liver in control male rats and animals subjected to 90-day dark deprivation (constant illumination). Data are presented as arithmetic mean ± standard deviation (M ± SD). Statistical significance of differences between the control and deprived groups in males is indicated by asterisks: * *p* < 0.05; ** *p* < 0.01; *** *p* < 0.001 (Welch’s *t*-test with Benjamini–Hochberg correction). The parameters are grouped into eight groups.

#### 3.1.1. Anthropometric Parameters

Dark deprivation did not affect body weight, but absolute liver weight significantly increased by 14.7%. Relative liver weight under dark deprivation also increased by 14.3% (Table 1).

#### 3.1.2. Micromorphometric Parameters of Hepatocytes

Hepatocyte area under dark deprivation increased by 40.5%. The nuclear area remained unchanged, while the nuclear–cytoplasmic ratio significantly decreased, indicating cytoplasmic hypertrophy (Table 2).

#### 3.1.3. Ploidy, Binucleated Cells, and Proliferative Activity Under Dark Deprivation

Constant illumination caused a decrease in ploidy by 11.2% and a reduction in the proportion of binucleated hepatocytes by 37.2%. Proliferative activity (Ki-67) increased by 34.2% (Table 3).

#### 3.1.4. Metabolic Parameters

Glycogen content in hepatocytes under dark deprivation decreased by 18.1%, while lipid content increased by 29.2%. Serum glucose increased by 15.6%. Total protein and albumin decreased by 10.7% and 15.3%, respectively (Table 4).

#### 3.1.5. Markers of Cellular Senescence, Apoptosis, and Tissue Entropy

Dark deprivation causes the most pronounced changes in senescence markers. p16 increased by 23.8 times, p21 by 6.7 times, and p53 increased by 80.9%. Entropy increased by 21.4% (Table 5).

#### 3.1.6. Circadian Genes and Melatonin

Constant illumination caused a profound suppression of Bmal1 expression—a 3.7-fold decrease, and a 3.5-fold decrease in Clock. Per2 levels, conversely, increased by 29.8%. Serum melatonin concentration decreased more than twofold (Table 6).

#### 3.1.7. Mitochondrial Parameters

Three-week dark deprivation caused a decrease in mitochondrial density by 13.1%. Mitochondrial area remained unchanged. The number of cristae decreased by 7.3%, and the circularity index significantly decreased, indicating an impairment of mitochondrial shape (Table 7).

#### 3.1.8. Biochemical Markers of Damage and Histological Scores

Under the influence of constant illumination, ALT levels slightly decreased, whereas AST increased by 27.2%. The steatosis score increased by 0.86 points. The pathological changes score increased by 3.1 times. However, total and direct bilirubin remained unchanged (Table 8).

### 3.2. Correlation and Regression Analyses of Entropy with Morphofunctional Parameters

#### 3.2.1. Correlations of Entropy with Other Parameters in Males

To assess the integrative role of entropy as a marker of structural tissue disorganization, a Pearson correlation analysis was performed between entropy and 33 morphofunctional parameters in rats (*n* = 120). Correction for multiple comparisons was carried out using the Benjamini–Hochberg method (FDR); correlations with q < 0.05 were considered statistically significant.

The table reflects the correlational relationships of entropy, as a measure of disorganization/chaos in the system, with a broad spectrum of morphological, functional and biochemical liver markers. For all parameters except the last two (histological scales), the Pearson correlation coefficient was used (*n* = 120). For the two ordinal damage scales, Spearman’s rank correlation was used. The significance level was adjusted for multiple comparisons (q < 0.05 considered statistically significant).

Very strong positive correlations of entropy (r > 0.85, q → 0) were found with indices of fatty dystrophy, optical density of lipids in hepatocytes, hepatocyte area, and the proportion of p53-positive cells. Strong negative correlations (r < −0.7) were found with melatonin level, Bmal1 and Clock expression, albumin level, and total protein.

Moderate and weak but still significant correlations of entropy were detected with the nuclear–cytoplasmic ratio (N/C ratio), mitochondrial circularity index, ALT, the proportion of binucleated hepatocytes, and mitochondrial density.

##### Partial Correlations Adjusted for Treatment

To clarify the independent associations of entropy not driven solely by the dark deprivation itself, partial correlations were calculated (controlling for the variable “treatment”). The results are presented in Table 9.

After adjustment for the experimental treatment, tissue entropy retained pronounced positive associations with most morphological, cellular, metabolic and ultrastructural liver parameters. The strongest partial correlations were detected with fatty dystrophy, ploidy, the proportion of binucleated hepatocytes, lipid and glycogen optical density, as well as with indices of mitochondrial ultrastructure. At the same time, anthropometric measures and serum biochemical markers of liver injury showed no significant independent associations with entropy. These results indicate that entropy predominantly reflects local tissue-level, cellular and ultrastructural remodelling of the liver.

#### 3.2.2. Exploratory Multivariable Model of Tissue Entropy in Rats

To describe the multivariable structure of associations with tissue entropy, an exploratory multiple linear regression model was constructed. Shannon entropy was used as the dependent variable. Candidate predictors included parameters that showed significant univariable associations with entropy, as well as anthropometric variables. Stepwise model selection was performed using the Akaike information criterion.

The final model included five variables: fatty degeneration score, Per2-positive cells, Ki-67-positive cells, Bmal1-positive cells, and the nuclear-to-cytoplasmic ratio. The model showed high apparent explanatory ability, explaining 87.5% of the variance in entropy.

Four of the five variables included in the final model were statistically significantly associated with entropy after correction for multiple testing: fatty degeneration score, Per2-positive cells, Ki-67-positive cells, and the nuclear-to-cytoplasmic ratio. Bmal1-positive cells remained in the AIC-selected model but did not reach statistical significance as an individual predictor.

At the same time, variance inflation factors were markedly elevated for all predictors, indicating substantial multicollinearity. Therefore, the model should be interpreted primarily as an exploratory multivariable model describing the combined association of several interrelated morphofunctional parameters with entropy. Individual regression coefficients should not be interpreted as fully independent or causally separable effects.

The final regression equation is:

H = 3.288 + 0.862 × Fatty degeneration score − 0.055 × Per2-positive cells, % + 0.783 × Ki-67-positive cells, % + 0.008 × Bmal1-positive cells, % − 4.706 × Nuclear-to-cytoplasmic ratio

where H is the entropy value.

Table 10 and Table 11 presents the regression coefficients, their standard errors, 95% confidence intervals, and significance levels.

The model explained 87.5% of the variance in tissue entropy. Four of the five variables included in the final model were statistically significant after correction for multiple testing. However, all predictors demonstrated high variance inflation factors, indicating pronounced multicollinearity. Therefore, although the model had high explanatory capacity, its coefficients should be interpreted with caution. The results support the integrative character of tissue entropy but do not allow reliable separation of the independent contribution of each individual predictor.

Model diagnostics

The main assumptions of linear regression were tested:

- Normality of residuals: the Shapiro–Wilk test gave *p* = 0.680, which does not reject the hypothesis of a normal distribution of residuals.
- Homoscedasticity: the Breusch–Pagan test (χ^2^ = 5.651, *p* = 0.342) showed no significant heteroscedasticity.
- Linearity: partial residual plots for each predictor revealed no systematic deviations from linearity.
- Independence of residuals: the Durbin–Watson statistic was 2.130 (*p* = 0.400), indicating no autocorrelation.

**Figure 1:**
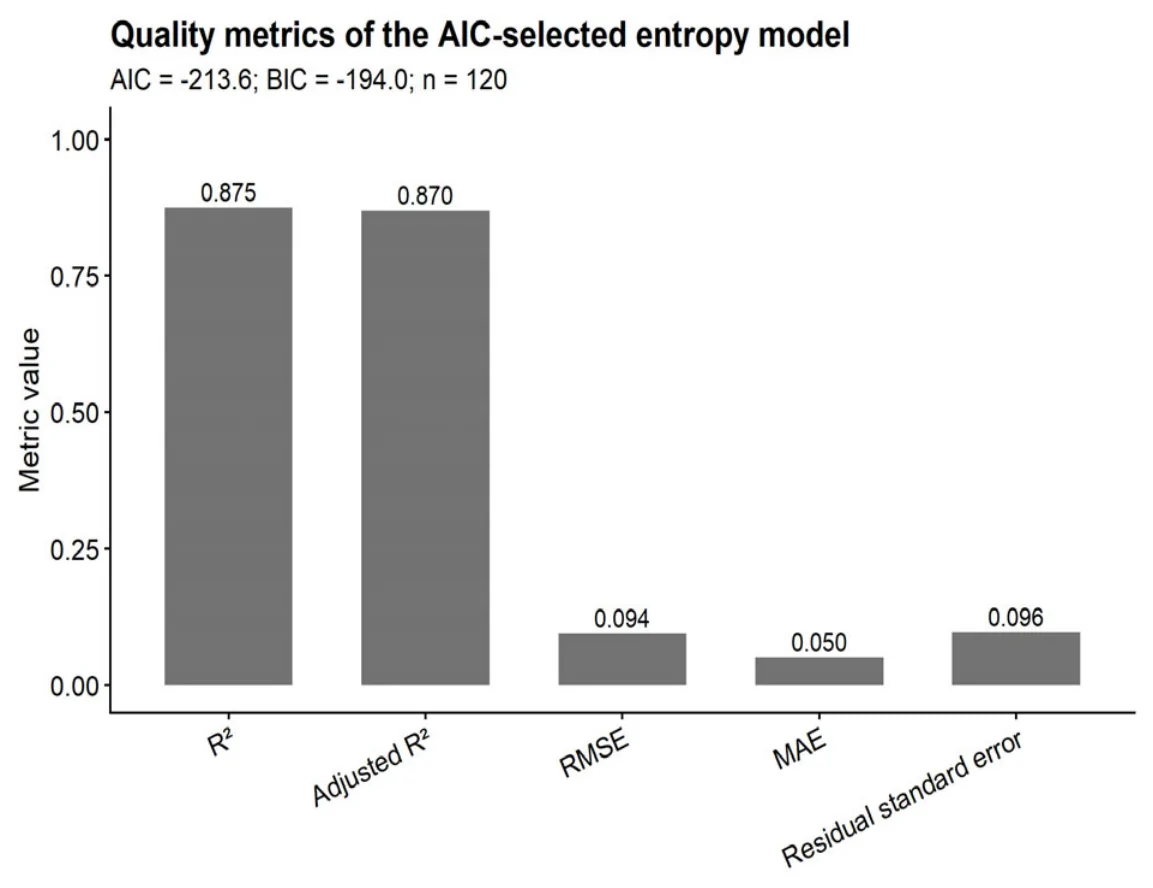
Entropy regression model performance.

Figure 1 shows a plot of predicted versus observed entropy values, demonstrating good model fit.

## 4. Discussion

### 4.1. Overview of Findings

The present study demonstrates that prolonged constant light exposure in rats is associated with a complex of morphofunctional changes that overlap with those observed in aging: hepatomegaly, steatosis, cellular senescence (p16, p21, p53), circadian gene dysregulation (BMAL1/CLOCK suppression, PER2 upregulation), melatonin reduction, mitochondrial dysfunction, and hyperglycemia. Structural entropy, calculated as a graph-based Shannon entropy from H&E-stained sections, was significantly increased in the LL group and showed strong correlations with most of these parameters. Partial correlation analyses, adjusted for treatment, indicated that entropy primarily reflects local tissue-level remodelling rather than systemic metabolic shifts. An exploratory regression model, albeit with substantial multicollinearity, suggested that entropy integrates multiple features of hepatic remodelling, with steatosis and circadian dysregulation as major contributors.

Importantly, these findings are correlational and hypothesis-generating. The study lacked naturally aged comparator groups, melatonin-replacement groups, recovery groups, and independent validation cohorts. Therefore, while the observed phenotype shares multiple features with aging, we cannot conclude that constant light exposure causes true accelerated aging, nor that entropy is a validated biomarker of biological liver age. Rather, this work provides a foundation for future investigations with appropriate experimental designs.

### 4.2. Constant Light Exposure and Hepatic Remodelling

Chronic disruption of circadian rhythms by constant light exposure is increasingly recognized as a risk factor for metabolic and age-associated disorders [11,38,39]. In rodents, prolonged circadian disruption has been associated with shortened lifespan and the appearance of signs resembling accelerated aging [40]. In our model, 3-month constant light exposure was associated with a more than twofold reduction in serum melatonin, accompanied by suppression of Bmal1 and Clock expression (3.7- and 3.5-fold, respectively) and a 29.8% increase in Per2-positive cells. This pattern is consistent with desynchronization of central and peripheral oscillators, in which hepatic Per2 may be upregulated as a compensatory or dysregulated response.

However, it is important to recognize that photic cues are among the most powerful entraining agents for circadian rhythms. Light signals act not only via the pineal–melatonin axis but also through direct retinohypothalamic projections to the suprachiasmatic nucleus, which in turn modulates autonomic nervous system outflow to peripheral organs, including the liver [12,13]. Constant illumination likely disrupts multiple pathways simultaneously. Therefore, while melatonin suppression is a prominent feature of our model, the overall phenotype likely results from combined circadian desynchronization, autonomic imbalance, and metabolic dysregulation, rather than from melatonin deficiency alone. Owing to the absence of a melatonin-supplemented control group, we cannot draw any definitive conclusions about the causal role of melatonin in the observed effects. This is a critical limitation that must be addressed in future studies.

The observed changes in circadian gene expression were accompanied by marked hepatocyte hypertrophy (cell area increased by 40.5%, nuclear-to-cytoplasmic ratio decreased by 30.4%), which is a feature commonly observed in the aging liver [15]. The reduction in ploidy (11.2%) and binucleated hepatocytes (37.2%) further supports the notion of altered hepatocellular differentiation and regeneration, which are also characteristic of aged livers [28]. These morphological changes, together with the metabolic alterations (hyperglycaemia, reduced albumin and total protein, increased lipids), suggest that constant light exposure induces a state of hepatic stress that phenocopies certain features of physiological aging.

### 4.3. Cellular Senescence and Mitochondrial Dysfunction

The most pronounced differences between groups were observed in senescence markers. p16-positive cells increased 23.8-fold, p21 increased 6.7-fold, and p53 increased by 80.9% in the LL group compared with controls. Hepatocyte senescence is now viewed as a dynamic continuum in which p21 may reflect reversible stress, whereas p16 marks a more irreversible senescent state [41]. The simultaneous sharp increase in both markers in our model suggests accelerated accumulation of senescent cells, consistent with the idea that chronodisruption promotes cellular ageing.

The observed suppression of melatonin, along with disruption of central and peripheral circadian oscillators, was accompanied by mitochondrial deterioration: mitochondrial density decreased by 13.1%, cristae number by 7.3%, and the circularity index (reflecting organelle shape) decreased by 13.2%. These findings are consistent with the known role of melatonin in mitochondrial protection, as reported in previous studies [42,43]. Melatonin is a powerful mitochondrially targeted antioxidant, and its decline has been associated with mitochondrial dysfunction and steatosis in the aging liver [44]. However, because we did not include a melatonin-replacement group, we cannot attribute these effects solely to melatonin deficiency. Constant light exposure affects multiple photic-entrained pathways, which may independently contribute to mitochondrial impairment.

Thus, constant light exposure, via chronodisruption and associated metabolic disturbances, appears to trigger a cascade leading to mitochondrial insufficiency and lipid accumulation—two hallmarks of aging-associated hepatic pathology. However, direct evidence for accelerated biological aging requires comparison with naturally aged animals, which was beyond the scope of this study.

### 4.4. Structural Entropy as an Exploratory Index of Tissue Disorganization

A key finding of this study is that structural entropy, calculated as graph-based Shannon entropy on standard H&E-stained sections, increased by 21.4% in the LL group and correlated strongly with most parameters studied. The strongest positive associations were observed with fatty degeneration score (r = 0.93), lipid optical density (r = 0.91), the proportion of p53-positive cells (r = 0.88), and the proportion of Per2-positive cells (r = 0.86). Strong negative correlations were found with melatonin level (r = −0.81) and expression of Bmal1/Clock (r ≈ −0.76). However, these unadjusted correlations reflect both between-group differences and within-group variability; partial correlations adjusted for treatment were performed to disentangle these effects.

After adjustment for treatment, entropy retained strong positive associations with morphological and ultrastructural parameters (fatty degeneration, ploidy, proportion of binucleated hepatocytes, lipid and glycogen optical density, mitochondrial indices) but lost significant associations with serum biochemical markers (albumin, total protein, AST, ALT, glucose) and melatonin. This suggests that entropy primarily reflects local tissue-level and cellular remodelling rather than systemic metabolic shifts. This is an important distinction: entropy appears to capture structural disorganization at the tissue level independently of the global metabolic disturbances induced by constant light exposure.

Melatonin is a powerful mitochondrially targeted antioxidant, and its age-related decline contributes to the development of steatosis and mitochondrial dysfunction of the liver [44]. In our study, the fall in melatonin was accompanied by a deterioration of mitochondrial parameters: mitochondrial density decreased by 13.1%, cristae number by 7.3%, and the circularity index, which reflects organelle shape, decreased by 13.2%. These data are in good agreement with work demonstrating that melatonin preserves mitochondrial ultrastructure and prevents steatosis in the aging liver [43,44,45,46]. Thus, constant light, via melatonin suppression, triggers a cascade leading to mitochondrial insufficiency and lipid accumulation—two key hallmarks of organ aging 

However, our data do not prove that entropy is a biomarker of biological age. Rather, they demonstrate that entropy is associated with a constellation of aging-associated morphological and molecular changes. Whether entropy increases with natural aging, predicts functional decline, or responds to geroprotective interventions requires direct testing in aged animals, longitudinal studies, and interventional designs. At present, entropy should be viewed as a promising exploratory index of tissue disorganization that warrants further investigation.

The concept of structural entropy is readily integrable into modern digital pathology workflows. Entropy indices can be fully automated and computed on routine H&E stains, without the need for additional immunohistochemical or biochemical tests [47]. The integrative nature of entropy enables it to capture the combined effect of multiple pathogenetic pathways (steatosis, senescence, mitochondrial dysfunction), which is particularly valuable for monitoring interventions targeting circadian rhythms and metabolism [48,49]. However, before entropy can be recommended for clinical or preclinical use, rigorous validation in independent cohorts and against established aging biomarkers is required.

### 4.5. Exploratory Regression Model: Descriptive, Not Predictive

The exploratory regression model, which included fatty degeneration score, the proportion of Per2-, Ki-67-, and Bmal1-positive cells, and the nuclear-to-cytoplasmic ratio, explained 87.5% of the variance in entropy in the current dataset. The dominant contribution of steatosis (standardized coefficient β = 1.50) suggests that lipid accumulation is a major driver of structural disorganization in this model. The negative contribution of Per2 may reflect its recently described anti-lipogenic role [50]. However, several caveats must be emphasized.

First, the high R^2^ was obtained on the same dataset used for model development, which is a known source of optimistic bias. Independent validation in a separate cohort is required before any predictive claims can be made. Second, variance inflation factors were markedly elevated (VIF up to 240), indicating severe multicollinearity among predictors. Therefore, individual regression coefficients cannot be interpreted as independent or causal effects of specific variables. Third, the model was constructed using AIC-based stepwise selection, which is sensitive to minor changes in the dataset. The inclusion or exclusion of specific predictors should not be overinterpreted.

Thus, the model should be interpreted as an exploratory description of the association structure, not as a validated predictive or causal model. Despite these limitations, the model supports the hypothesis that entropy integrates multiple features of hepatic remodelling—steatosis, circadian dysregulation, proliferative response, and cellular architecture—consistent with its proposed role as an integrative index of tissue disorganization.

### 4.6. Comparison with Previous Studies

Our findings are consistent with previous work on the effects of constant light exposure on the liver. Grabeklis et al. [14] demonstrated that constant illumination causes reorganization of circadian rhythms of morphofunctional liver parameters. Ogrodnik et al. [15] showed that cellular senescence and mitochondrial dysfunction are key features of age-related liver disease. Our study extends these observations by demonstrating that these changes occur in concert with increased structural entropy, and that entropy correlates with multiple hallmarks of aging-associated hepatic pathology.

The concept of structural entropy has been previously applied in pathology, particularly in the context of neoplasms [20,21,22,23]. Kayser and colleagues demonstrated that entropy measures can characterize tissue heterogeneity in cancers of the breast, prostate, bladder, and lung [22]. Our study extends this approach to the context of chronodisruption and aging-associated hepatic remodelling, suggesting that entropy may have broader applications beyond oncology.

However, our study differs from previous work in that we explicitly acknowledge the exploratory nature of our findings and the need for independent validation. While previous studies have often presented entropy as a validated biomarker, we take a more cautious approach, recognizing the limitations of our study design.

### 4.7. Future Directions

The present study is hypothesis-generating rather than confirmatory. To establish whether constant light truly accelerates liver aging and whether entropy is a valid biomarker of biological age, the following experiments are required:

1. **Comparison with naturally aged rats** (e.g., 18- and 24-month-old animals) to determine whether the entropy increase and senescence marker profile in LL-exposed young rats quantitatively or qualitatively match those of physiological aging.
2. **Recovery groups** to assess reversibility of entropy and other parameters after restoration of normal photoperiod. If entropy decreases after recovery, this would suggest that the observed changes are reversible and reflect adaptive remodelling rather than permanent aging.
3. **Melatonin supplementation and receptor knockout models** to dissect the causal role of melatonin versus other light-entrained pathways (e.g., autonomic nervous system). A group with exogenous melatonin administration under constant light (LL + Melatonin) would allow us to determine whether melatonin deficiency is a necessary mediator of the observed effects.
4. **Independent validation cohort** to test the predictive value of entropy for aging-related outcomes. This could involve a separate set of animals with different ages or treatment conditions.
5. **Longitudinal sampling** to establish the trajectory of entropy changes over time. Cross-sectional data cannot distinguish between transient stress responses and progressive aging.
6. **Molecular validation by qPCR** to confirm the observed changes in senescence and circadian gene expression at the transcriptional level.
7. **Measurement of pro-inflammatory cytokines** (IL-6, TNF-α, HMGB1) to directly assess the involvement of the SIRT1/HMGB1/NF-κB axis in the observed phenotype.

Until such studies are performed, entropy should be viewed as a promising exploratory index rather than a validated biomarker. The present work provides a foundation and rationale for these future investigations.

## 5. Limitations

The following limitations should be considered when interpreting the results of this study:

**Absence of naturally aged comparator group.** Our study included only young adult rats (3–6 months) exposed to constant light, without a group of naturally aged animals (e.g., 18- and 24-month-old rats). Therefore, we cannot directly compare the magnitude or trajectory of changes with physiological aging. The term “aging-associated” is used to indicate phenotypic resemblance, not proven acceleration of the biological aging process.

**Lack of melatonin replacement group.** The strong inverse correlation between entropy and serum melatonin (r = −0.81) does not prove causality. To establish the causal role of melatonin, an additional group with exogenous melatonin administration under constant light (LL + Melatonin) is necessary. Our study design does not allow us to determine whether the observed effects are specifically due to melatonin deficiency or to broader chronodisruption.

**Lack of recovery group.** Without a group in which animals are returned to a normal 12:12 h light/dark cycle after constant light exposure, we cannot determine whether the observed changes are reversible or permanent. This distinction is critical for evaluating the potential for chronobiological interventions.

**No molecular validation by qPCR.** Although we assessed protein expression by immunohistochemistry, quantitative PCR for senescence and circadian genes (e.g., *Cdkn2a/p16*, *Cdkn1a/p21*, *Trp53/p53*, *Bmal1*, *Clock*, *Per2*) would strengthen the conclusions at the transcriptional level.

**Inflammatory markers not directly measured.** We did not measure pro-inflammatory cytokines (IL-6, TNF-α, HMGB1) in liver tissue or serum, so the involvement of the senescence-associated secretory phenotype (SASP) and the SIRT1/HMGB1/NF-κB axis remains inferred rather than directly demonstrated.

**Species and sex specificity.** All animals were male Wistar rats, a nocturnal species with circadian physiology that differs significantly from diurnal humans. Extrapolation to humans and to females requires caution. Sex differences in circadian rhythms, melatonin metabolism, and susceptibility to liver diseases are well established; our findings may not generalise to females.

**2D versus 3D entropy.** Our entropy measure is based on 2D nuclear arrangements in histological sections. Two-dimensional analysis inherently cannot capture the full 3D architectural complexity of the liver. Advances in 3D histology and tissue clearing techniques may provide more accurate entropy estimates in future work.

**Methodological considerations for entropy calculation.** The entropy measure used in this study is a graph-based entropy derived from nuclear spatial distribution and size heterogeneity. While this approach is well-validated conceptually and has shown strong correlations with multiple functional parameters, it requires careful nuclear segmentation and filtering. Variability in segmentation algorithms could affect entropy values, although we performed robustness checks with different bin numbers and edge weighting schemes.

**Correlational design.** This is a correlational study, not a mechanistic or interventional study. All associations reported are correlational and do not imply causation. The exploratory regression model, while demonstrating high apparent fit, is descriptive and not validated for prediction.

**Single time point.** The study included only one time point (3 months). Longitudinal data would be required to establish the temporal sequence of changes and to determine whether entropy changes precede or follow functional decline.

## 6. Conclusions

Constant light exposure induces a complex of changes in rats that mirror aging-associated phenotypes: hepatomegaly, steatosis, hyperglycaemia, accumulation of senescent cells (p16, p21, p53), suppression of circadian genes (Bmal1, Clock), a paradoxical increase in Per2, reduced melatonin levels, and mitochondrial dysfunction.

Structural entropy of histological images, calculated as graph-based Shannon entropy on H&E-stained sections, increased significantly during constant light exposure and correlated with key aging-associated markers. Partial correlation analyses, adjusted for treatment, indicated that entropy reflects local tissue-level and cellular remodelling independently of systemic metabolic shifts. The exploratory regression model, while limited by multicollinearity and the lack of independent validation, supported the hypothesis that entropy integrates multiple features of hepatic remodelling, with steatosis and circadian dysregulation as major contributors.

However, entropy should be viewed as an exploratory quantitative index of tissue disorganization rather than a validated biomarker of biological liver age. Its potential as a geroprotective screening tool and its applicability in digital pathology require independent validation in aged animals, recovery models, melatonin-replacement studies, and longitudinal designs. The present work provides a foundation and rationale for such future investigations.

We conclude that structural entropy is a promising integrative measure of aging-associated hepatic disorganization that warrants further study. Its computational simplicity and applicability to routine histological sections make it an attractive candidate for digital pathology applications, provided that rigorous validation is performed in appropriately designed studies.

## Figures and Tables

**Table 1:** Body weight, absolute and relative liver weight of rats.

Outcome	Control	Constant Light	Mean/Median Difference	Effect Size	Statistic	*p*	q
Body weight, g	345.60 ± 11.70	346.79 ± 11.73	1.20 [−3.04 to 5.43]	0.10 [−0.25 to 0.46]	0.577	0.581	0.581
Absolute liver weight, g	11.13 ± 0.37	12.77 ± 0.43	1.64 [1.49 to 1.78]	4.05 [3.42 to 4.67]	1.12 × 10^−43^	3.47 × 10^−43^	3.47 × 10^−43^
Relative liver weight, %	3.22 ± 0.01	3.68 ± 0.01	0.46 [0.46 to 0.46]	55.91 [48.76 to 63.05]	7.47 × 10^−173^	2.54 × 10^−171^	2.54 × 10^−171^

**Table 2:** Nuclear area, hepatocyte area, and nuclear–cytoplasmic ratio.

Outcome	Control	Constant Light	Mean/Median Difference	Effect Size	Statistic	*p*	q
Nuclear area, µm^2^	42.37 ± 3.66	43.11 ± 3.17	0.74 [−0.50 to 1.98]	0.21 [−0.14 to 0.57]	1.18	*p* = 0.241	q = 0.273
Hepatocyte area, µm^2^	189.84 ± 14.39	266.58 ± 20.59	76.74 [70.31 to 83.17]	4.29 [3.64 to 4.95]	23.67	*p* = 5.25 × 10^−44^	q = 1.78 × 10^−43^
Nuclear-to-cytoplasmic ratio	0.24 ± 0.02	0.16 ± 0.02	−0.07 [−0.08 to −0.06]	−3.75 [−4.35 to −3.16]	−20.69	*p* = 1.95 × 10^−40^	q = 5.09 × 10^−40^

**Table 3:** Ploidy, proportion of binucleated hepatocytes, and Ki-67 proliferation index.

Outcome	Control	Constant Light	Mean/Median Difference	Effect Size	Statistic	*p*	q
Ploidy, 2c units	4.76 ± 0.82	4.22 ± 0.56	−0.53 [−0.79 to −0.28]	−0.75 [−1.12 to −0.39]	−4.16	*p* = 6.58 × 10^−5^	q = 8.29 × 10^−5^
Binucleated hepatocytes, %	7.69 ± 1.60	4.83 ± 0.97	−2.86 [−3.34 to −2.38]	−2.16 [−2.60 to −1.71]	−11.88	*p* = 1.24 × 10^−20^	q = 2.00 × 10^−20^
Ki-67-positive cells, %	1.01 ± 0.17	1.36 ± 0.24	0.35 [0.27 to 0.42]	1.68 [1.26 to 2.09]	9.25	*p* = 2.56 × 10^−15^	q = 3.96 × 10^−15^

**Table 4:** Glycogen and lipid content in hepatocytes, serum glucose, total protein, and albumin.

Outcome	Control	Constant Light	Mean/Median Difference	Effect Size	Statistic	*p*	q
Glycogen optical density	0.30 ± 0.05	0.25 ± 0.02	−0.06 [−0.07 to −0.04]	−1.56 [−1.97 to −1.16]	−8.62	*p* = 3.93 × 10^−13^	q = 5.56 × 10^−13^
Lipid optical density	0.32 ± 0.04	0.41 ± 0.02	0.09 [0.08 to 0.10]	2.75 [2.25 to 3.25]	15.15	*p* = 2.59 × 10^−24^	q = 4.90 × 10^−24^
Glucose, mmol/L	7.14 ± 0.23	8.25 ± 0.20	1.12 [1.04 to 1.19]	5.14 [4.40 to 5.89]	28.34	*p* = 3.50 × 10^−54^	q = 1.98 × 10^−53^
Total protein, g/L	68.58 ± 2.39	61.23 ± 1.93	−7.35 [−8.14 to −6.57]	−3.37 [−3.92 to −2.81]	−18.56	*p* = 4.20 × 10^−36^	q = 9.52 × 10^−36^
Albumin, g/L	34.56 ± 1.44	29.28 ± 1.20	−5.28 [−5.76 to −4.80]	−3.95 [−4.56 to −3.33]	−21.75	*p* = 1.91 × 10^−42^	q = 5.40 × 10^−42^

**Table 5:** Expression of p53, p16, p21, and entropy value.

Outcome	Control	Constant Light	Mean/Median Difference	Effect Size	Statistic	*p*	q
p53-positive cells, %	2.20 ± 0.27	3.99 ± 0.43	1.78 [1.65 to 1.91]	4.89 [4.17 to 5.61]	26.97	*p* = 2.53 × 10^−47^	q = 9.56 × 10^−47^
p16-positive cells, %	0.50 ± 0.10	12.01 ± 1.68	11.51 [11.07 to 11.94]	9.60 [8.32 to 10.88]	52.92	*p* = 1.06 × 10^−51^	q = 5.15 × 10^−51^
p21-positive cells, %	1.11 ± 0.14	7.40 ± 1.04	6.29 [6.02 to 6.56]	8.41 [7.28 to 9.54]	46.35	*p* = 2.17 × 10^−49^	q = 9.20 × 10^−49^
Shannon entropy	1.99 ± 0.20	2.42 ± 0.10	0.43 [0.37 to 0.49]	2.69 [2.20 to 3.19]	14.84	*p* = 2.42 × 10^−25^	q = 4.85 × 10^−25^

**Table 6:** Expression of Bmal1, Clock, Per2, and serum melatonin concentration.

Outcome	Control	Constant Light	Mean/Median Difference	Effect Size	Statistic	*p*	q
Bmal1-positive cells, %	60.79 ± 2.28	16.44 ± 1.95	−44.35 [−45.12 to −43.58]	−20.74 [−23.41 to −18.07]	−114.30	1.90 × 10^−120^	3.24 × 10^−119^
Clock-positive cells, %	54.73 ± 2.13	15.64 ± 1.93	−39.09 [−39.82 to −38.35]	−19.11 [−21.58 to −16.65]	−105.35	1.06 × 10^−117^	1.20 × 10^−116^
Per2-positive cells, %	31.04 ± 2.10	40.28 ± 3.69	9.24 [8.15 to 10.32]	3.06 [2.53 to 3.58]	16.85	4.22 × 10^−30^	8.97 × 10^−30^
Serum melatonin, pg/mL	17.57 ± 1.10	8.49 ± 0.69	−9.08 [−9.41 to −8.75]	−9.83 [−11.13 to −8.52]	−54.16	1.12 × 10^−75^	9.49 × 10^−75^

**Table 7:** Density, area, perimeter, number of cristae, and circularity index of mitochondria.

Outcome	Control	Constant Light	Mean/Median Difference	Effect Size	Statistic	*p*	q
Mitochondrial density, n/µm^2^	1.69 ± 0.14	1.47 ± 0.15	−0.22 [−0.27 to −0.17]	−1.53 [−1.94 to −1.13]	−8.45	*p* = 9.00 × 10^−14^	q = 1.33 × 10^−13^
Mitochondrial area, µm^2^	0.32 ± 0.02	0.32 ± 0.06	0.01 [−0.01 to 0.02]	0.13 [−0.22 to 0.49]	0.73	*p* = 0.469	q = 0.515
Mitochondrial perimeter, µm	1.95 ± 0.09	2.00 ± 0.08	0.04 [0.01 to 0.08]	0.48 [0.12 to 0.84]	2.64	*p* = 0.009	q = 0.011
Cristae count	23.52 ± 1.96	21.81 ± 2.48	−1.71 [−2.52 to −0.90]	−0.76 [−1.13 to −0.39]	−4.17	*p* = 5.94 × 10^−5^	q = 7.77 × 10^−5^
Area-to-perimeter ratio	0.23 ± 0.02	0.24 ± 0.03	0.01 [0.00 to 0.02]	0.57 [0.21 to 0.93]	3.14	*p* = 0.002	q = 0.003
Circularity index	1.08 ± 0.03	0.94 ± 0.04	−0.14 [−0.16 to −0.13]	−3.81 [−4.41 to −3.21]	−21.00	*p* = 6.11 × 10^−38^	q = 1.48 × 10^−37^

**Table 8:** Transaminase activity, bilirubin, and steatosis/pathological changes scores.

Outcome	Control	Constant Light	Mean/Median Difference	Effect Size	Statistic	*p*	q
ALT, U/L	60.53 ± 2.35	58.62 ± 2.38	−1.90 [−2.76 to −1.05]	−0.80 [−1.17 to −0.43]	−4.40	*p* = 2.40 × 10^−5^	q = 3.27 × 10^−5^
AST, U/L	122.89 ± 5.31	156.25 ± 5.09	33.36 [31.48 to 35.24]	6.37 [5.48 to 7.26]	35.11	*p* = 3.06 × 10^−64^	q = 2.08 × 10^−63^
Total bilirubin, µmol/L	30.49 ± 2.02	30.26 ± 1.76	−0.23 [−0.91 to 0.46]	−0.12 [−0.48 to 0.24]	−0.66	*p* = 0.512	q = 0.544
Direct bilirubin, µmol/L	10.82 ± 0.72	10.75 ± 0.66	−0.07 [−0.32 to 0.18]	−0.10 [−0.46 to 0.26]	−0.55	*p* = 0.581	q = 0.581
Fatty degeneration score	0.20 [0.05; 0.40]	1.06 [0.92; 1.18]	0.86	0.99	3574.00	*p* = 1.18 × 10^−20^	q = 2.00 × 10^−20^
Pathological changes score	0.12 [0.11; 0.14]	0.38 [0.37; 0.40]	0.26	1.00	3600.00	*p* = 2.84 × 10^−21^	q = 5.09 × 10^−21^

**Table 9:** Partial correlations between entropy and morphofunctional markers adjusted for treatment.

Domain	Marker	Method	*n*	Partial r	95% CI	*p*	q
Anthropometry	Body weight, g	Partial Pearson	120	−0.14	−0.31 to 0.04	*p* = 0.131	q = 0.232
	Absolute liver weight, g	Partial Pearson	120	−0.13	−0.30 to 0.05	*p* = 0.156	q = 0.232
	Relative liver weight, %	Partial Pearson	120	0.11	−0.07 to 0.28	*p* = 0.232	q = 0.232
Hepatocyte morphometry	Ploidy, 2c units	Partial Pearson	120	0.76	0.67 to 0.82	*p* = 3.22 × 10^−23^	q = 8.39 × 10^−23^
	Binucleated hepatocytes, %	Partial Pearson	120	0.76	0.67 to 0.82	*p* = 3.36 × 10^−23^	q = 8.39 × 10^−23^
	Nuclear area, µm^2^	Partial Pearson	120	0.71	0.61 to 0.79	*p* = 1.69 × 10^−19^	q = 2.82 × 10^−19^
	Nuclear-to-cytoplasmic ratio	Partial Pearson	120	0.69	0.59 to 0.78	*p* = 2.09 × 10^−18^	q = 2.62 × 10^−18^
	Hepatocyte area, µm^2^	Partial Pearson	120	0.65	0.53 to 0.74	*p* = 2.40 × 10^−15^	q = 2.40 × 10^−15^
Cell proliferation and senescence	Ki−67-positive cells, %	Partial Pearson	120	0.64	0.52 to 0.74	*p* = 4.79 × 10^−15^	q = 1.91 × 10^−14^
	p53-positive cells, %	Partial Pearson	120	0.62	0.49 to 0.72	*p* = 8.33 × 10^−14^	q = 1.67 × 10^−13^
	p21-positive cells, %	Partial Pearson	120	0.34	0.17 to 0.49	*p* = 1.64 × 10^−4^	q = 2.18 × 10^−4^
	p16-positive cells, %	Partial Pearson	120	0.31	0.14 to 0.46	*p* = 6.01 × 10^−4^	q = 6.01 × 10^−4^
Circadian axis	Bmal1-positive cells, %	Partial Pearson	120	0.70	0.59 to 0.78	*p* = 8.45 × 10^−19^	q = 3.38 × 10^−18^
	Clock-positive cells, %	Partial Pearson	120	0.69	0.58 to 0.78	*p* = 2.95 × 10^−18^	q = 5.90 × 10^−18^
	Per2-positive cells, %	Partial Pearson	120	0.58	0.44 to 0.69	*p* = 6.84 × 10^−12^	q = 9.12 × 10^−12^
	Serum melatonin, pg/mL	Partial Pearson	120	−0.14	−0.31 to 0.04	*p* = 0.137	q = 0.137
Metabolic profile	Lipid optical density	Partial Pearson	120	0.75	0.66 to 0.82	*p* = 5.64 × 10^−23^	q = 2.82 × 10^−22^
	Glycogen optical density	Partial Pearson	120	0.75	0.65 to 0.82	*p* = 2.49 × 10^−22^	q = 6.24 × 10^−22^
	Glucose, mmol/L	Partial Pearson	120	−0.09	−0.27 to 0.09	*p* = 0.322	q = 0.448
	Albumin, g/L	Partial Pearson	120	−0.08	−0.26 to 0.10	*p* = 0.393	q = 0.448
	Total protein, g/L	Partial Pearson	120	−0.07	−0.25 to 0.11	*p* = 0.448	q = 0.448
Serum biochemistry	AST, U/L	Partial Pearson	120	−0.09	−0.26 to 0.09	*p* = 0.342	q = 0.453
	Total bilirubin, µmol/L	Partial Pearson	120	−0.08	−0.25 to 0.10	*p* = 0.405	q = 0.453
	Direct bilirubin, µmol/L	Partial Pearson	120	−0.07	−0.25 to 0.11	*p* = 0.452	q = 0.453
	ALT, U/L	Partial Pearson	120	−0.07	−0.25 to 0.11	*p* = 0.453	q = 0.453
Mitochondrial ultrastructure	Mitochondrial perimeter, µm	Partial Pearson	120	0.68	0.58 to 0.77	*p* = 8.89 × 10^−18^	q = 5.34 × 10^−17^
	Mitochondrial density, n/µm^2^	Partial Pearson	120	0.66	0.55 to 0.75	*p* = 1.77 × 10^−16^	q = 5.32 × 10^−16^
	Cristae count	Partial Pearson	120	0.64	0.51 to 0.73	*p* = 8.59 × 10^−15^	q = 1.72 × 10^−14^
	Area-to-perimeter ratio	Partial Pearson	120	0.59	0.46 to 0.70	*p* = 1.38 × 10^−12^	q = 2.07 × 10^−12^
	Circularity index	Partial Pearson	120	0.59	0.45 to 0.69	*p* = 2.79 × 10^−12^	q = 3.34 × 10^−12^
	Mitochondrial area, µm^2^	Partial Pearson	120	0.51	0.36 to 0.63	*p* = 4.43 × 10^−9^	q = 4.43 × 10^−9^
Histological injury	Fatty degeneration score	Partial Spearman	120	0.77	0.69 to 0.84	*p* = 9.99 × 10^−25^	q = 2.00 × 10^−24^
	Pathological changes score	Partial Spearman	120	0.75	0.65 to 0.82	*p* = 2.45 × 10^−22^	q = 2.45 × 10^−22^

**Table 10:** Exploratory multivariable linear regression model for tissue entropy in male rats.

Predictor	B	SE	β Standardized	95% CI	t	*p*	q	Partial R^2^	VIF
Intercept	3.288	0.411	—	2.473 to 4.103	7.99	*p* = 1.19 × 10^−12^	—	—	—
Fatty degeneration score	0.862	0.138	1.499	0.589 to 1.135	6.25	*p* = 7.32 × 10^−9^	q = 3.66 × 10^−8^	0.255	52.58
Per2-positive cells, %	−0.055	0.019	−1.137	−0.092 to −0.018	−2.91	*p* = 0.004	q = 0.011	0.069	139.49
Ki-67-positive cells, %	0.783	0.345	0.786	0.100 to 1.466	2.27	*p* = 0.025	q = 0.042	0.043	109.40
Bmal1-positive cells, %	0.008	0.006	0.711	−0.004 to 0.021	1.39	*p* = 0.169	q = 0.169	0.017	240.83
Nuclear-to-cytoplasmic ratio	−4.706	2.234	−0.718	−9.132 to −0.280	−2.11	*p* = 0.037	q = 0.047	0.037	106.06

**Table 11:** Model characteristics.

Metric	Value
*n*	120
Predictors	5
R^2^	0.875
Adjusted R^2^	0.870
RMSE	0.094
MAE	0.050
Residual standard error	0.096
F statistic	159.96 (df = 5; 114)
Model *p*-value	*p* = 8.20 × 10^−50^
AIC	−213.6
BIC	−194.0

## Data Availability

The original contributions presented in this study are included in the article. Further inquiries can be directed to the corresponding author.
